# A Fusion Between Common Bean Phaseolin and the N‐Terminal Domain of Maize 16 kDa Gamma Zein: Clues for Designing Nutritionally Improved Seed Storage Proteins

**DOI:** 10.1111/pbi.70719

**Published:** 2026-07-05

**Authors:** Emma Costa, Lucrezia Luciani, Mahsa Taghipour Rahimi, Alessandro Vitale, Emanuela Pedrazzini

**Affiliations:** ^1^ CNR Institute of Agricultural Biology and Biotechnology Milano Italy

**Keywords:** maize prolamins, protein intracellular traffic, protein nutritional improvement, recombinant fusion proteins, seed storage proteins, zeolin

## Abstract

Increasing the nutritional value of seed storage proteins (SSPs) has been largely hindered by limited knowledge of how the features of the various SSP domains determine high accumulation in protein storage vacuoles (PSV) or as endoplasmic reticulum (ER)‐located insoluble protein bodies (PB). We had previously designed zeolin, a fusion between phaseolin (PHSL, the main bean SSP, a soluble trimeric protein located in PSV) and the N‐terminal domain of 27 kDa γ‐zein (27γz, a major maize PB prolamin). Zeolin has a more balanced essential amino acid composition than each parental sequence and forms insoluble homomeric PB very similar to natural maize heteropolymeric PB, indicating that the zein domain has a dominant effect over PHSL. We had also determined that the paralog 16 kDa γ‐zein (16γz) remains partially soluble and forms unusual threads in the ER when ectopically expressed. We now produced zeolin2 (zeo2), by fusing PHSL to the N‐terminal domain of 16γz. We show that only a minor proportion of zeo2 remains in the ER, without forming PBs or threads. Zeo2 mainly forms soluble trimers with biochemical characteristics very similar to PHSL trimers, and traffics to the vacuole via the Golgi complex. This indicates that the 16γz domain fails to significantly dominate over PHSL. In transgenic arabidopsis, zeo2 accumulates to markedly higher levels than phaseolin and only slightly lower levels than zeolin. Zeo2 is also more accessible than zeolin to proteases during the early stages of seed germination. These findings provide new information relevant for the design of modified, nutritionally enhanced storage proteins.

## 1 Introduction

During their development, seeds store large amounts of a few unique protein classes to be used as an amino acid source during early germination. Because of their abundance, seed storage proteins (SSPs) constitute a large part of the human protein diet, with wheat being the single main source of protein for human nutrition worldwide (Burlingame et al. 2024). SSPs can be divided into three classes: monomeric 2S albumins, trimeric/hexameric 7S/11S globulins, and the highly polymeric prolamins (Yang et al. 2023). All SSPs belong to the very large group of secretory proteins, which are co‐translationally inserted into the endoplasmic reticulum (ER) lumen. Members of the first two classes are widespread among plants, traffic from the ER along the secretory pathway, and accumulate in the specialized seed storage vacuoles (Zheng et al. 2022). Prolamins are instead unique to grasses and in many cases are stored as large insoluble heteropolymers, which form round‐shaped structures termed protein bodies (PBs) in the ER lumen (Pedrazzini et al. 2016). Despite these unusual properties (the ER has evolved to be a protein nursery, not a protein storage compartment), most prolamins are evolutionarily linked to the soluble 2S albumins, originating by deletions that destroy their disulphide bond‐mediated conformation or by insertions of new domains containing cysteine residues and repeats with amphipathic or hydrophobic properties (Xu and Messing 2009; Pedrazzini et al. 2016).

Increasing the nutritional value of SSPs has been a major goal of agricultural genetics for decades, but it has been largely hampered by a limited understanding of how the specific structural properties characterizing different domains of SSPs drive the processes that allow for their accumulation in seed cells. Besides their agricultural and nutritional importance, the peculiar characteristics of prolamins have stimulated research on whether they can be used in helping the production of recombinant proteins for various purposes (Torrent et al. 2009; Arcalis et al. 2014; Schwestka et al. 2023), and to study the extent of cellular response to the accumulation of high amounts of protein in the ER lumen (Vitale and Pedrazzini 2022). This research has specially focussed on 27 kDa γ‐zein, one of the maize prolamins.

In maize, prolamins are encoded by around thirty genes that can be divided into four subclasses: α‐, β‐, δ‐ and γ‐zeins (Holding 2014). 27 kDa γ‐zein (27γz), the major maize prolamin, is constituted by an N‐terminal domain with eight repeated amphipathic hexapeptides and seven Cys residues, followed by a 2S albumin‐like C‐terminal domain (Prat et al. 1985). 27γz, which is insoluble in physiological conditions but is solublized in buffers containing reducing agents, is the major constituent of the PB layer which surrounds the large PB core mainly formed by α‐zeins and is in contact with the lumenal side of the ER membrane (Lending and Larkins 1989). 27γz is one of the first zeins to be synthesized during seed development (Lending and Larkins 1989), initiating PB formation and forms homotypic PBs when expressed individually in vegetative tissues of transgenic plants (Geli et al. 1994). The N‐terminal domain is sufficient to promote efficient PB formation also when fused to the C‐terminus of the 7S *Phaseolus vulgaris* storage protein phaseolin (the construct was named zeolin), confirming its dominant effect suggested by the 27γz structure (Mainieri et al. 2004). The Cys residues in the N‐terminal domain are determinant for 27γz insolubility and its retention in the ER lumen: when all seven residues were mutated to Ser, 27γz was efficiently secreted (Mainieri et al. 2014). Progressive deletion of the amphipathic repeats in a construct consisting of the N‐terminal domain followed by a fluorescent protein indicates that these are also important for PB formation (Llop‐Tous et al. 2010).

The typical maize genome contains three γ‐zein genes, encoding polypeptides of 27, 50 and 16 kDa. The latter, quantitatively a minor zein with an as yet unclear role in PB formation, has appeared more recently, probably during the maize whole genome duplication followed by gene deletion events (Xu and Messing 2008). The major structural differences between 16γz and 27γz are in the N‐terminal domain: most of the amphipathic repeats and three out of the seven Cys residues are missing in 16γz (Prat et al. 1987; Mainieri et al. 2018). Unlike 27γz, 16γz is not fully solubilized by reducing agents, suggesting stronger interactions with the alcohol‐soluble α‐zeins present in the PB core (Mainieri et al. 2018). When expressed ectopically in vegetative plant tissues, 16γz forms disulphide‐bonded polymers retained in the ER, but fails to assemble into PBs, forming instead unusual extended threads that markedly enlarge the ER lumen (Mainieri et al. 2018). Given these characteristics, in an effort to create neoproteins with optimized nutritional value and to test the possible biotechnological applications of 16γz, we have determined in this study the behaviour of a new zeolin form, named zeolin2 (zeo2), in which the 27γz domain was replaced by that of 16γz.

## 2 Material and Methods

### 2.1 Plasmid Construction and *Agrobacterium tumefaciens Transformation*

*A. tumefaciens* strain *GV3101 (pSOUP)* transformed with *pGA470‐zeolin* plasmid, containing the zeolin‐coding sequence, has been described in Mainieri et al. 2004. To construct the zeo2 expression plasmid, a SpeI/SphI restriction fragment containing the last 402 bp coding for the C‐terminal region of phaseolin, followed by 45 bp coding for the (GGGGS)3 linker peptide and 273 bp coding for the mature N‐terminal region of 27 kDa γ‐zein, was excised from the *pDHA‐zeolin* plasmid (see Mainieri et al. 2004) and replaced with a SpeI/SphI synthetic fragment coding for the same portion of the excised phaseolin sequence and linker peptide followed by 141 bp coding for the N‐terminal domain of 16 kDa γ‐zein without the signal peptide. The resulting plasmid was named *pDHA‐zeolin2*. The EcoRI fragment containing the entire expression cassette, including the regulatory elements (CaMV 35S promoter and terminator), was excised from *pDHA‐zeolin2* and subcloned into the EcoRI‐linearized *pGreenII0179* binary vector (http://www.pgreen.ac.uk, John Innes Centre, Norwich, Norfolk, UK) carrying the kanamycin and hygromycin resistances for agrobacterium and plant selection, respectively. The resulting *pGreenII::zeolin2* construct was introduced into the *Agrobacterium tumefaciens* strain *GV3101 (pSOUP)*, which has been used for transient and stable expression experiments.

### 2.2 Transient Expression in *Nicotiana tabacum* Leaves

*N. tabacum* (cv. Petite Havana SR1) plants, wild type (wt) or constitutively expressing the ER‐localized fluorescent protein GFP‐HDEL (Foresti et al. 2003), were cultivated in soil in a growth chamber at 25°C with a 16 h/8 h light/dark cycle. For transient expression, leaves from 4 to 6‐week‐old wt or GFP‐HDEL plants were infiltrated with *Agrobacterium tumefaciens GV3101* strain carrying the *pGreenII::zeolin* or *pGreenII::zeolin2* plasmids, growing at an optical density of 0.2. The agroinfiltrated plants were then irrigated to restore turgor pressure and incubated for 2 to 6 days at 25°C in the growth chamber. Two days after infiltration, small sections of GFP‐HDEL infiltrated leaves were placed on a microscope slide and visualized with a 63× objective mounted on an Axiovert 200 microscope (Carl Zeiss, Oberkochen, Germany) equipped for epifluorescence. GFP‐HDEL fluorescence is detected at 488 nm/520 nm and 543 nm/616 nm excitation/emission.

After 2, 3 or 6 days, leaf pieces of wt tobacco infiltrated with zeolin or zeo2 agrobacteria or mock infiltrated were frozen in liquid nitrogen and stored at −80°C for subsequent biochemical analyses.

### 2.3 Stable Expression in *Arabidopsis thaliana*

Transgenic *Arabidopsis thaliana* (ecotype Columbia) plants expressing zeolin or zeo2 were produced by the floral dipping method (Clough and Bent 1998) using the transformed *A. tumefaciens* described above. Hygromycin resistant T0 plants were identified and the homozygous progenies were selected. Plants were grown in soil at 21°C/23°C under a 16 h/8 h light/dark cycle or in sterile conditions on half‐concentrated Murashige and Skoog media (Duchefa Biochemie) supplemented with 10 g/L Sucrose and 0.8% (w/v) phyto agar (Duchefa Biochemie) and 25 mg/mL hygromycin. Experiments were then conducted using T3 or T4 plants.

### 2.4 Protein Analysis

Agroinfiltrated *N. tabacum* leaf fragments (wt or GFP‐HDEL) or transgenic *A. thaliana* leaves were homogenized in a mortar on ice with a ratio 1:7 weight/volume of homogenization buffer (HB: 150 mM NaCl, 1.5 mM EDTA, 1.5% Triton X‐100, 150 mM Tris‐Cl, pH 7.8, cOmplete Protease Inhibitor Cocktail [Roche]) supplemented with 4% 2‐Mercaptoethanol. Homogenates were centrifuged at 1000 g, 4°C, 10 min to remove the debris and supernatant used for further analysis. For the solubility assay, leaves were homogenized in HB in the absence of 2‐Mercaptoethanol, and soluble and insoluble proteins were separated by centrifugation at 1500 g, 10 min, 4°C. Samples were then adjusted to 1.0% SDS, 4% 2‐ME and analysed by 15% SDS‐PAGE followed by protein blot probed with the appropriate antibodies and detection with SuperSignal West Pico Chemiluminescent substrate (Thermo Scientific, Rockford, USA). Unstained Protein MW Marker (Fermentas, Vilnius, Lithuania) was used as molecular mass markers.

### 2.5 Antibodies

The following antisera or antibodies were used, at the indicate dilutions. Rabbit polyclonal anti‐phaseolin (1:5000, Pedrazzini et al. 1997); rabbit polyclonal anti‐tobacco BIP (1:10000 Pedrazzini et al. 1997); goat anti‐rabbit IgG‐peroxidase conjugate (1:16000, Pierce Biotechnology Rockford, IL, USA).

### 2.6 Digestion With Endoglycosidase H (Endo H) or Peptide N‐Glycanase F (PNGase F)

Leaf homogenate from arabidopsis Col‐0 or transgenic plants containing 30 μg of total proteins were denatured in 1× final Glycoprotein Denaturing Buffer (stock 10×, New England Biolabs, Beverly, MA, USA) for 10 min at 100°C. G5 buffer (for EndoH) or G7 buffer (for PNGaseF) were added to 1× final (stock 10X for both the buffers). For PNGase F, 1% NP‐40 (10% stock) was also added because the activity of this enzyme is inhibited by SDS. Samples were split into three aliquots and subjected to digestion with 2000 units of Endo Hf (1000 U/μL, New England Biolabs) or 1000 units PNGase F (1,000 U/μL, New England Biolabs), or mock digested, using the reagents and the protocol supplied by the manufacturer. After 1 h of incubation at 37°C, the reactions were stopped by adding Laemmli denaturation buffer.

Proteins were analysed by 15% SDS‐PAGE followed by protein blotting as described above.

### 2.7 Trypsin Digestion

Total leaf homogenates from zeo2, zeolin or PHSL transgenic plants were centrifuged at 600 g for 5 min at 4°C in order to remove the starch that could interfere with the digestion. 200 μL of clarified homogenates were supplemented with 10 μL of trypsin (from a 10 mg/mL stock solution prepared in 1 mM HCl) or with 10 μL of 1 mM HCl as a control. Samples were then incubated at 37°C for 0, 15, 30 min. At each time point, 60 μL aliquots were taken, immediately supplemented with cOmplete Protease Inhibitor Cocktail [Roche] and brought to 0°C to stop the reaction. Equal amounts of samples were then denatured and analysed by SDS‐PAGE followed by western blot incubated with anti‐PHSL antibodies.

### 2.8 In Vivo Treatment With Brefeldin A or Dithiothreitol

Seedlings from zeolin or zeo2 transgenic plants were incubated for 24 h in the presence (+BFA) or absence (‐BFA) of 15 μg/mL brefeldin A. After homogenization, equal volumes of samples were analysed by SDS‐PAGE and protein blot using anti‐PHSL antiserum. Total proteins were stained with Ponceau S, for normalization and as loading control. Intact zeolin or zeo2 polypeptides and PHSL fragments were quantified by densitometry of their specific protein blot bands using Image Lab software (Bio‐Rad Laboratories S.r.l.). Protein amounts were calculated relative to the filter backgrounds and normalized on the amount of RuBisCO Large subunit (RbcL), The ratio of intact zeolin or zeo2 polypeptides to phaseolin fragments were calculated in two independent experiments (exp 1, exp. 2).

For dithiothreitol (DTT) treatment, seedlings from zeo2, zeolin or PHSL transgenic plants were incubated for 24 h with or without 2 mM DTT in water. Proteins were extracted and equal amounts were analysed by SDS‐PAGE and protein blot using anti‐PHSL antiserum. The PHSL fragments in zeolin, zeo2 or PHSL samples were quantified by densitometry of their specific protein blot bands (using Image Lab software, Bio‐Rad Laboratories S.r.l.), relative to the filter background, and expressed as percentage of the total amount of the detected polypeptides in the lane. The fold change in the percentages of PHSL fragments in DTT‐treated on the untreated samples (% + DTT/% −DTT) were calculated. Data are from three fully independent biological replicates. Statistical significance was determined by one‐way ANOVA followed by post hoc Tukey HSD test (significance level *p* < 0.05). Values with different letters are significantly different (*p* < 0.05).

### 2.9 Subcellular Fractionation on Isopycnic Sucrose Gradients

Young leaves of transgenic and control plants were homogenized in the absence of detergent with a 7:1 (v/w) ratio of 100 mM Tris‐Cl pH 7.8, 10 mM KCl, 12% sucrose (w/w), Complete protease inhibitor cocktail buffer, containing either 1 mM EDTA or 10 mM MgCl_2_. Six hundred microliters of each sample were loaded on the top of 12 mL of liner sucrose gradient [100 mM Tris‐Cl pH 7.8, 10 mM KCl, 16%–65% sucrose (w/w)] and centrifuged at 150000 g for 2 h at 4°C in a Beckman SW40 rotor (Beckman, Fullerton, CA, USA). After centrifugation, 18 fractions of about 700 μL were collected and equal amounts of each fraction were denatured and analysed by SDS‐PAGE and protein blot. The distribution in gradient fractions of different polypeptides detected by anti‐PHSL antibodies were quantified by densitometry of their specific protein blot bands and expressed as percentage (%) of the sum of all the detected polypeptides in the relative blots.

### 2.10 Velocity Centrifugation on Sucrose Gradient

Young leaves of transgenic and Col‐0 arabidopsis plants were homogenized in HB with or without 4% 2‐Mercaptoethanol. The homogenate under non‐reducing conditions was loaded onto a linear gradient from 5% to 25% (w/v) sucrose prepared in 150 mM NaCl, 1 mM EDTA, 0.1% Triton X‐100, 50 mM Tris‐Cl, pH 7.5. The homogenate obtained under reducing conditions was loaded onto a similar gradient supplemented with 2 mM dithiothreitol (DTT). An additional gradient prepared without DTT was loaded with a mixture of protein markers containing 200 μg each of cytochrome C (12.4 kDa), ovalbumin (43 kDa), BSA (66 kDa), aldolase (161 kDa) and catalase (232 kDa). After centrifugation at 200000 g for 20 h at 4°C in a Beckman SW40 rotor, 17 fractions of about 750 μL were collected. An equal volume of each fraction was analysed by SDS‐PAGE and protein blot. Zeo2 different assembly forms in fractions from oxidizing or reducing gradients were quantified by densitometry of their specific protein blot bands and expressed as percentage (%) of the sum of all the detected forms in the relative blots.

### 2.11 Time‐Course Degradation During Germination

Twenty dry seeds from zeolin or zeo2 transgenic arabidopsis were time‐course germinated in water in the presence of 100 mM cycloheximide to inhibit new polypeptide synthesis. At each time‐point, germinating seeds were homogenized in a mortar on ice with 80 μL of 10 mM KCl, 2 mM MgCl_2_, 100 mM Tris‐Cl pH 7.8 and cOmplete Protease Inhibitor Cocktail [Roche]. Equal volume of each homogenate was analysed by SDS–PAGE, followed by protein blot with anti‐PHSL antibodies. Total proteins were stained with Ponceau S. The total amounts of intact zeolin and zeo2 polypeptides were quantified by densitometry of their specific protein blot bands, normalized to the amounts of RuBisCO Large subunit (RbcL), and expressed as percentage (%) relative to the amounts at 0 h = 100%. Data are from four independent biological replicates and statistical significance was determined by pairwise *T*‐test with post hoc Tukey HSD test at each time point and the significance level was set to *p* < 0.05 (*p < 0.05; **p < 0.01).

### 2.12 Transmission Electron Microscopy

TEM analysis was performed at the UNITECH NO LIMITS core facility infrastructure at the University of Milan, Italy (https://www.unimi.it/en/research/places‐organizations‐and‐infrastructures/unitech/nolimits‐unitech). Three‐week‐old leaves from Col‐0, zeolin or zeo2 plants were fixed in 2.5% glutaraldehyde, 4% paraformaldehyde in 0.1 M sodium cacodylate buffer, pH 7.4 for 4 h at room temperature. Samples were rinsed 3 times for 10 min with sodium cacodylate buffer 0.1 M, dissected into small pieces (1 mm × 1 mm) and post‐fixed in 1% osmium tetroxide in 0.1 M cacodylate buffer for 2 h at 4°C. After washes in 0.1 M cacodylate buffer pH 7.4 and water, samples were counter‐stained with 0.5% uranyl acetate in water overnight at 4°C in the dark. After washes, samples were dehydrated through ethanol series (with the last twice dehydration in 100% ethanol and propylene oxide) and progressively infiltrated with a mixture of Epon‐Araldite:propylene‐oxide. Samples were then embedded in pure resin before polymerization at 60°C for 48 h. Resin‐embedded samples were cut with the ultramicrotome (PowerTome XL, RMC) into semi‐thin sections of 0.5 μm thickness, stained with 0.1% toluidine blue in 0.1 M sodium phosphate buffer, and observed at the optical microscope to identify the region of the sample to investigate at the ultrastructural level. Ultra‐thin sections of 70 nm were cut with a diamond knife (Ultra 35°, DIATOME) and collected on copper grids (G200‐Cu, Electron Microscopy Sciences). Samples were observed at the transmission electron microscope (TEM) (Talos L120C, Thermo Fisher Scientific) at 120 kV, and images were acquired with a digital camera (Ceta CMOS Camera, Thermo Fisher Scientific).

## 3 Results

### 3.1 Zeolin2 Does Not Form High‐Density PBs and Undergoes Markedly Increased Traffic to the Vacuole Compared to Zeolin

**FIGURE 1:**
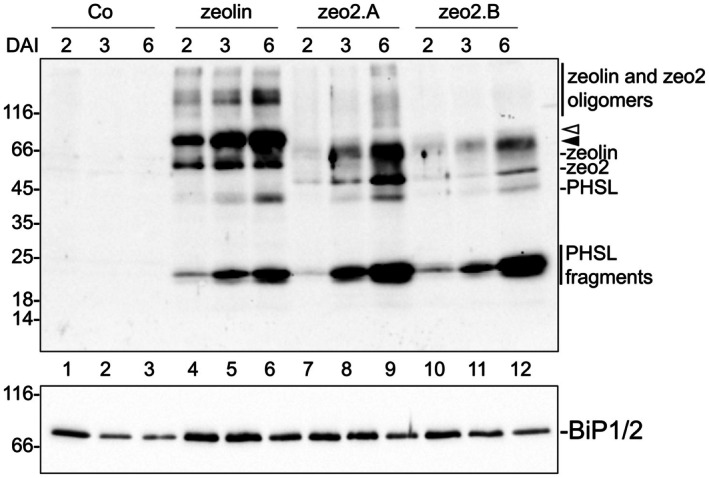
Transient expression by agroinfiltration of *N. tabacum* leaves, time course and BiP amounts. *N. tabacum* leaves were transiently transfected with agrobacteria carrying plasmids encoding zeolin (lanes 4–6) or zeo2 (lanes 7–12, two different plants, zeo2 A and zeo2 B) or empty plasmid (lanes 1–3, Co). Two, three and six days after infiltration, leaves were homogenized and equal amounts of extract were analysed by SDS‐PAGE followed by protein blot incubated with anti‐PHSL (top panel) or anti‐BiP (bottom panel) antibodies. The positions of zeolin or zeo2 monomers, oligomers, as well as of the released PHSL and PHSL fragments are indicated. White or black arrowheads: Zeolin and zeo2 forms, respectively, modified by proline hydroxylation followed by O‐glycosylation. Numbers on the left indicate the position and size (kDa) of molecular mass markers.

Zeolin contains the primary sequence of T343F phaseolin (termed PHSL hereon, for brevity), including its signal peptide for insertion into the ER lumen, and 89 amino acids of mature 27 kDa γ‐zein (27γz) starting from the fifth residue after the 27γz signal peptide (Mainieri et al. 2004). This 27γz fragment consists of the almost complete N‐terminal domain of the maize protein, including all the repeated Pro‐rich hexapeptides and six out of its seven Cys residues. The unstructured 15 amino acid linker (GGGGS) 3 was inserted between the two sequences, to favour their independent correct folding (Mainieri et al. 2004). An identical strategy was used to construct zeolin2 (Figure S1). The paralogous zeolin2 domain is composed of 46 amino acids only, which include the two degenerated Pro‐rich repeats and three out of the four N‐terminal Cys residues of 16γz. The nucleotide sequence encoding zeolin2 (hereafter referred to as zeo2) was placed under the control of the CaMV 35S constitutive promoter (the same used for zeolin expression) and was first tested by agroinfiltration in *Nicotiana tabacum* , using empty plasmid or plasmid expressing zeolin as controls. At 2, 3 or 6 days after infiltration (DAI), leaf tissue was homogenated without reducing agents. After solubilization by SDS in reducing conditions, total proteins were analysed by SDS‐PAGE followed by immunoblot with anti‐phaseolin (anti‐PHSL) antiserum (Figure 1). The most abundant products detected upon zeolin expression at 2 DAI migrated slightly above (white arrowhead) and below (zeolin) the 66 kDa molecular mass marker (Figure 1, upper panel, lane 4 and compare with lane 1). These are the already reported electrophoretic mobilities of modified and unmodified intact zeolin monomers detected in transgenic tobacco leaves (Mainieri et al. 2004). Modified zeolin most probably underwent prolyl hydroxylation followed by O‐glycosylation of a number of hydroxyproline residues when expressed in cells of vegetative tissues, as it has been observed for 27γz (Mainieri et al. 2004, 2014). Two less abundant components around 45 and 20 kDa, also observed when zeolin is expressed in tobacco, represent intact PHSL and PHSL fragments (Figure 1, upper panel), which are produced when the ectopically expressed bean storage protein traffics to vegetative vacuoles (Pedrazzini et al. 1997; Mainieri et al. 2004). We have previously hypothesized that the 45 and 20 kDa components originate from a minor proportion of zeolin polypeptides that fail to undergo polymerization into protein bodies, and trimerize because of the phaseolin portion of zeolin (Mainieri et al. 2004); these trimers remain soluble and are thus delivered to vacuoles, where proteases release phaseolin (45 kDa) and fragment it into two halves (Mainieri et al. 2004). At 3 and 6 DAI, modified zeolin, intact PHSL and its fragments increased in relative abundance with respect to unmodified zeolin, modified intact zeolin remaining the major detected form (Figure 1, upper panel, compare lanes 4, 5 and 6).

Expression of zeo2 (Figure 1, upper panel, lanes 7–9 and 10–12, representing two independent transfections of the same construct) also resulted in the accumulation of polypeptides consistent with the expected electrophoretic mobilities of the intact, unmodified (zeo2) or modified (black arrowhead) recombinant protein, with apparent mobilities between the 45 and 66 kDa markers, as well as intact PHSL and PHSL fragments. There is some variability in the relative amounts of intact zeo2 to PHSL fragments in different agroinfiltrated leaves; since the transient expression level of the recombinant protein is variable, this could be related to the known saturation of the vacuolar sorting machinery that can occur during transient high expression (Frigerio et al. 1998). However, PHSL fragments originating from zeo2 always represented the most abundant accumulated product, at all days analysed.

These results suggest that zeo2 mainly forms soluble polymers that traffic to the vacuole, being less efficient than zeolin in polymerizing into PB‐like insoluble structures unable to traffic along the secretory pathway.

The binding protein (BiP) is the ER‐located member of the heat‐shock 70 chaperone class, where it plays a major role in assisting proper folding of newly synthesized secretory polypeptides, targeting misfolded proteins to degradation and regulating the unfolded protein response (UPR), a cascade of signals that adjusts gene expression and protein synthesis to changes in the workload of the ER as a protein folding machinery (Ko and Brandizzi 2024). Consistently, BiP levels reflect the amount of ER workload or the synthesis of proteins with folding defects. Protein blot analysis with anti‐BiP antiserum showed an increase in BiP level upon the expression of both zeolin and zeo2 with respect to the control and PHSL, the effect of zeolin appearing stronger than that of zeo2 (Figure 1, bottom panel). These results suggest that zeo2 stresses the ER folding machinery to a lower extent than zeolin.

**FIGURE 2:**
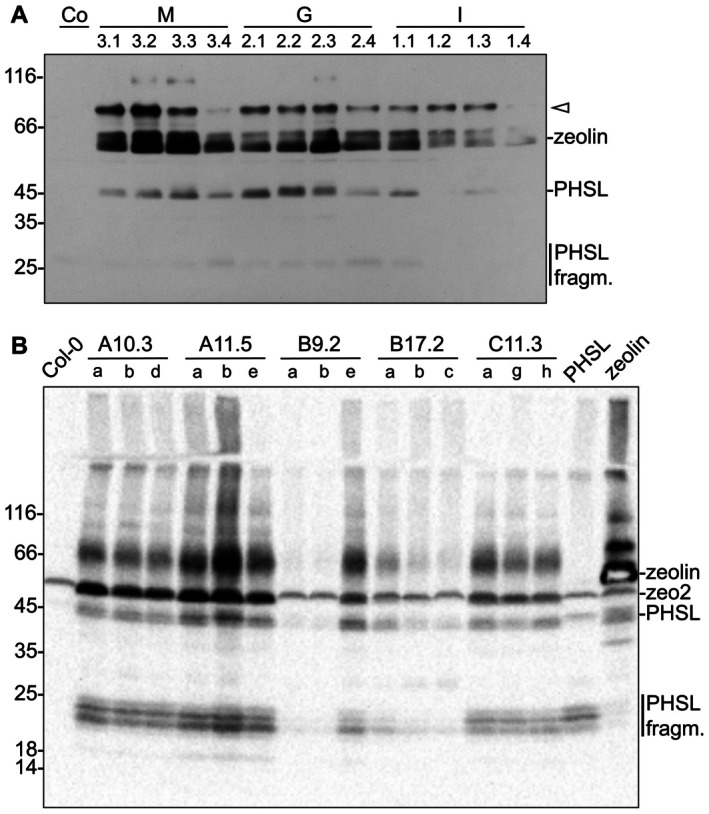
Zeolin and zeo2 in arabidopsis transgenic plants. (A) Equal amounts of leaf extracts from *A. thaliana* Col‐0 (Co) or different transgenic lines expressing zeolin by three independent transformation events (M, G, I) were subjected to SDS‐PAGE and protein blot analysis with anti‐PHSL antibodies. The positions of zeolin monomer, as well as of the released PHSL and PHSL fragments (PHSL fragm.) are indicated. White arrowhead: Zeolin modified by proline hydroxylation followed by O‐glycosylation. (B) Equal amounts of leaf extracts from representative plants (lower case letters) of different transgenic lines expressing zeo2 by three independent transformation events (A, B, C) were subjected to SDS‐PAGE and protein blot analysis with anti‐PHSL antibodies. The positions of zeo2 monomers, as well as of the released PHSL and PHSL fragments are indicated. Co, PHSL, zeolin: Leaf extract from Col‐0 or PHSL or zeolin transgenic plants. Numbers on the left in A and B: Position and size (kDa) of molecular mass markers.

The different behaviours of zeolin and zeo2 were confirmed by constitutive expression in transgenic *A. thaliana* plants. For each recombinant protein, the SDS‐PAGE banding patterns detected by anti‐PHSL antiserum were consistently reproduced in the analysis of leaf extracts from different plants derived by independent transgenic lines (Figure 2). A relatively very small amount of intact phaseolin and an even smaller amount of PHSL fragments, with respect to intact zeolin modified by proline hydroxylation followed by O‐glycosylation or unmodified, were detected upon zeolin expression (Figure 2A), consistently with previous results obtained by its expression in transgenic tobacco (Mainieri et al. 2004). The relative amounts of PHSL, and even more of its vacuolar degradation fragments, were much higher upon zeo2 expression, the ratio between intact PHSL and its fragments being very similar to the one observed when PHSL was expressed in transgenic arabidopsis (Figure 2B; notice that the high amount of accumulated intact zeolin oversaturates the signal). The ratio between released PHSL fragments and intact zeo2 is very similar in independent transgenic lines expressing zeo2 at different levels as in samples at different days after agroinfiltration, and is always much higher than the ratio observed when zeolin is expressed by agroinfiltration or in transgenic plants (Figures 1 and 2). This indicates that the formation or not of insoluble polymers depends on the structure and intrinsic features of the recombinant protein, and not on its expression levels.

The BiP level in zeo2, zeolin and PHSL transgenic seedlings in comparison to that in Col‐0 indicates that, while zeolin strongly stimulates BiP synthesis, zeo2, similarly to PHSL, leads to only a slight increase in the chaperone level (Figure S2), confirming the results of transient expression. This strongly suggests that zeo2 folding and assembly is rather rapid, like that of PHSL, thus not involving prolonged interaction with BiP.

**FIGURE 3:**
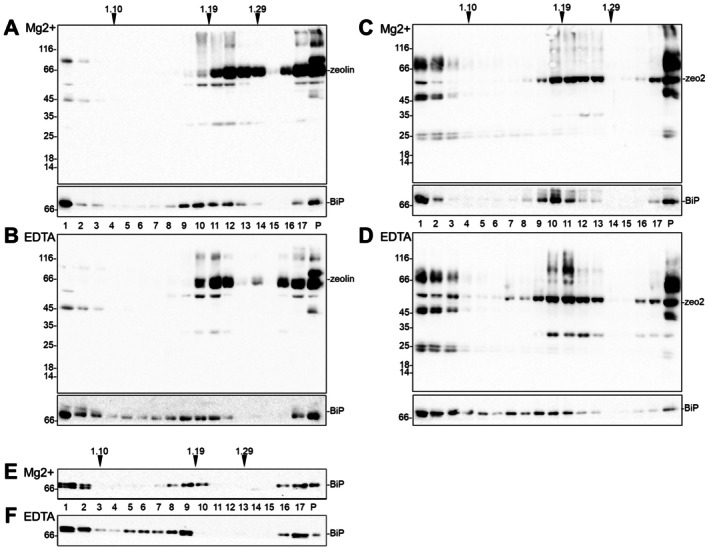
Zeo2 does not form high‐dense PBs, remaining available for intracellular traffic. Leaves from transgenic lines expressing zeolin (A, B), zeo2 (C, D) or Col‐0 plants (E, F) were homogenized in 12% (w/w) sucrose buffer in the absence of detergent, supplemented with MgCl_2_ or EDTA. The homogenates were fractionated by ultracentrifugation on isopycnic sucrose gradients containing MgCl_2_ (A, C, E) or EDTA (B, D, F), as indicated. The collected fractions were analysed by SDS‐PAGE, followed by immunoblotting with anti‐PHSL (top panels in A to D) or anti‐BiP (E, F and bottom panels in A to D) antisera. Numbers at top of A, C and E indicate sucrose density (g/mL). In each panel, numbers at left indicate the position and size (kDa) of molecular mass markers.

To determine the subcellular localization of intact zeo2 and to verify whether the higher accumulation of PHSL fragments was indeed due to traffic along the secretory pathway, leaf extracts prepared from zeo2 or zeolin plants in the absence of non‐ionic detergent were subjected to subcellular fractionation by isopycnic sucrose ultracentrifugation (Figure 3, and see Figure S3 for relative quantifications of the band intensities). When homogenation was performed in the presence of magnesium (Figure 3A, upper panel and S3A), intact unprocessed zeolin was recovered in gradient fractions towards the bottom of the tubes (fractions 16 to pellet), most probably mainly containing unbroken tissue, and in a clear high density peak up to 1.29 g/mL, very similar to PBs formed by 27γz in transgenic arabidopsis (Mainieri et al. 2018) and higher than the ER identified by anti‐BiP antiserum around 1.19 g/mL (Figure 3A, lower panel). Both zeolin and BiP peaks shifted to lower density when ribosomes were released from the ER membrane by replacing magnesium with the chelating agent EDTA (Figure 3B,S3B), confirming that PBs formed by zeolin in arabidopsis are surrounded by the rough ER membrane, like natural maize PBs (Lending and Larkins 1989). A small proportion of both modified zeolin and PHSL originating from zeolin migrated at the top of the gradients, where soluble vacuolar, cytosolic and apoplastic proteins are recovered, since vacuoles break during homogenation. Intact, unprocessed zeo2 was distributed in fractions with a density closer to that of the ER than to denser fractions containing zeolin PBs (Figure 3C,S3C). The zeo2 peak shifted to lower density in the presence of EDTA, but the shift was much less pronounced than that of zeolin PBs (Figure 3D, and compare BiP position in S3A‐D). A high proportion of modified zeo2, and the total amounts of PHSL and PHSL fragments were recovered at the top of gradients. A high proportion of BiP was also recovered in top fractions in any plant analysed, most probably reflecting partial release of this soluble chaperone from the ER during homogenation (Pedrazzini et al. 1997) or its traffic to vacuoles as a quality control process (Pimpl et al. 2006), but the fact that intact PHSL and PHSL fragments are totally absent from microsomal fractions indicates that a relevant proportion of zeo2 traffics out of the ER. Comparison with BiP in untransformed, control plants showed that the chaperone localization was not affected by zeo2 expression, but was in part shifted to denser ER fractions containing zeolin PBs (Figure 3E,F, compare with BiP in lower panels A and C, and see the corresponding quantifications in Figure S3). A polypeptide around 30 kDa, peaking in fractions also containing intact zeolin or zeolin2, is visible in Figure 3A–D. This polypeptide clearly includes part of the PHSL sequence, but its subcellular localization indicates that it is unlikely to be a product of vacuolar proteolysis.

The results of isopycnic fractionation indicate that zeo2 is not able to form highly dense PBs, is available for intracellular traffic in markedly higher proportion compared to zeolin and, at least in part, is delivered to the vacuole where the PHSL portion is fragmented by vacular proteases. The partial shift of BiP to higher density structures by zeolin but not zeo2 expression suggests that the latter has less extensive interaction with the chaperone and is consistent with its lower effect in stimulating BiP expression, shown in Figure 1 and Figure S2.

### 3.2 Traffic of a Relevant Proportion of Zeolin2 Is Golgi‐Mediated

**FIGURE 4:**
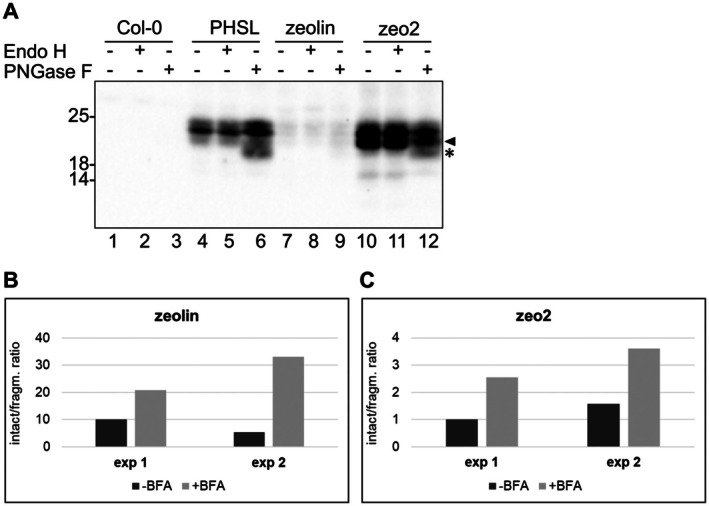
Zeo2 traffics to the vacuole via the Golgi complex. A: Leaf homogenates from Col‐0, PHSL, zeolin or zeo2 arabidopsis plants were incubated with the enzyme Endo H (+) or PNGase F (+), or without enzyme as control (−), and analysed by SDS‐PAGE followed by protein blot with anti‐PHSL antiserum. Asterisk and arrowhead indicate the positions of PNGase de‐glycosylated and Golgi‐modified glycosylated PHSL fragments, respectively. Numbers on the left indicate the position and size (kDa) of molecular mass markers. B and C: Seedlings from zeolin (B) or zeo2 (C) transgenic plants were incubated for 24 h in the presence (+BFA) or absence (−BFA) of 15 mg/mL brefeldin A. Proteins were extracted and equal volumes of homogenates were analysed by SDS‐PAGE and protein blot using anti‐PHSL antiserum. Intact zeolin or zeo2 polypeptides and PHSL fragments were quantified by densitometry. Protein amounts were calculated relative to the filter backgrounds and normalized on RbcL levels. The ratio of intact zeolin or zeo2 polypeptides to phaseolin fragments was calculated in two fully independent experiments (exp 1, exp. 2). Notice the different scales in panel B and C.

To determine whether indeed the relatively high amounts of PHSL fragments derived from zeo2 originated from Golgi‐mediated traffic to the vacuole, we first investigated the processing of its Asn‐linked oligosaccharide chain. The T343F PHSL sequence used to produce both zeolin and zeo2 contains the N‐glycosylation consensus site at Asn^252^, the second N‐glycosylation site (Asn^341^) having been inactivated by mutating the codon of Thr^343^ to a Phe codon to semplify the electrophoresis banding pattern (Pedrazzini et al. 1997). Asn^252^ is efficiently glycosylated in vivo, and its attached high‐mannose glycan is processed during PHSL traffic through the Golgi complex, becoming resistant to removal by endoglycosidase H (Sturm et al. 1987). Endoglycosydase H (Endo H) resistance can thus be used to verify whether the Golgi complex mediates the traffic of zeo2 through the secretory pathway. Asn^252^ is located in the smallest PHSL fragmentation product. Consistently with efficient Golgi‐mediated traffic, the banding pattern of the vacuolar fragmentation products of PHSL expressed in transgenic arabidopsis is not affected by endo H digestion, but a new fragment with higher mobility is produced by digestion with peptide‐N‐glycanase F (PNGase F), which is able to remove from glycoproteins both unprocessed and Golgi‐processed N‐linked glycans (Figure 4A, lanes 4–6). A similar new fragment is produced when zeo2 is digested with PNGase F, but not upon Endo H digestion (Figure 4A, lanes 7–9). Zeolin fragments are barely visible in this exposure of Figure 4A, lanes 7–9, but longer exposure shows the same pattern (Figure S4).

Golgi‐mediated traffic to vacuoles can be blocked by the fungal toxin brefeldin A (BFA), which therefore inhibits the fragmentation of PHSL in transgenic tobacco (Pedrazzini et al. 1997). We therefore expected that the generation of PHSL fragments from zeo2 would have been inhibited by BFA. Seedlings of zeo2 or zeolin plants were incubated for 24 h in the presence of BFA and the ratio between intact zeo2 or zeolin and PHSL fragments respectively originating from them was quantified (Figure 4B,C). As expected, in controls the ratio was much lower for zeo2 than zeolin (Figure 4B,C, black columns; notice the different scales in the two panels). In both cases, BFA caused a marked inhibition in the formation of PHSL fragments (grey columns).

From the results shown in Figure 4 we conclude that the PHSL fragments originate from both zeolin and zeo2 because of Golgi‐mediated traffic to vacuoles, and that zeo2 is much more competent than zeolin for this traffic.

### 3.3 Zeo2 Polymerizes Less Extensively Than Zeolin and Slightly Alters ER Morphology

**FIGURE 5:**
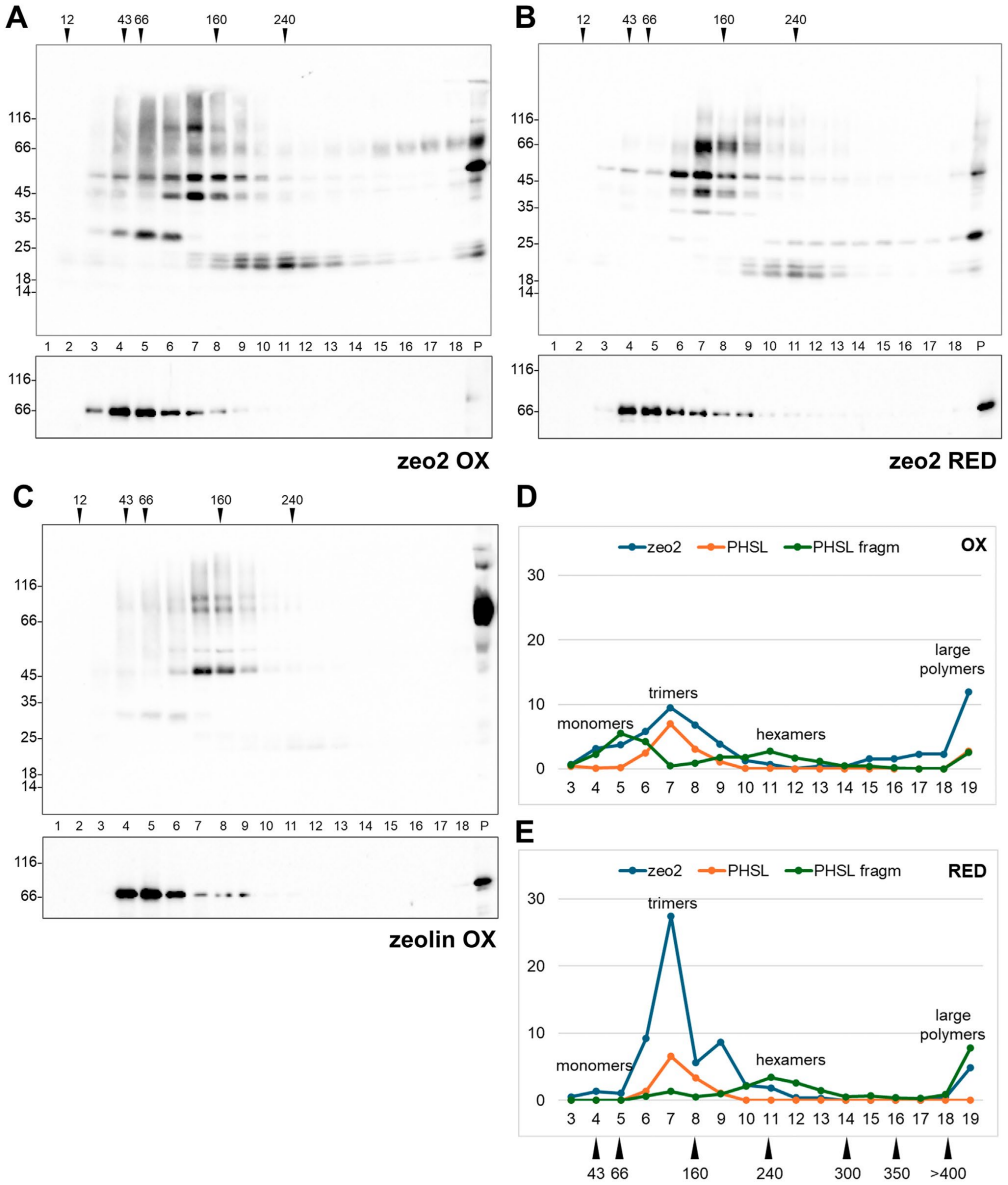
Oligomeric state of zeo2. Young leaves from transgenic arabidopsis plants expressing zeolin or zeolin 2 were homogenated in the absence or presence of 2‐ME. The homogenates were subjected to sedimentation velocity centrifugation on a continuous 5% to 25% (w/v) sucrose gradient supplemented (B, zeo2 RED) or not (A, zeo2 OX; C, zeolin 2 OX) with 2 mM DTT. Equal amounts of each fraction and a proportional amount of the pellet recovered at the bottom of the tubes (P) were analysed by protein blots incubated with anti‐PHSL (top panels) or anti‐BiP antisera (bottom panels). Numbers on the left: SDS‐PAGE molecular mass markers (kDa). Zeo2 different assembly forms in fractions from oxidizing or reducing gradients were quantified by densitometry of their specific protein blot bands and expressed as percentage (%) of the sum of all the detected forms in the relative blots (panels D and E, respectively). Numbers on the top in A‐C and on the bottom in D‐E: Position and molecular mass (kDa) of sedimentation markers. Numbers on the left in D and E: Percentage of protein (%).

When ectopically expressed, both 27γz and 16γz assemble into very large polymers that remain in the ER and migrate at the bottom of tubes upon velocity ultracentrifugation (Mainieri et al. 2004, 2018). In both cases, polymerization is mainly due to inter‐chain disulphide bonds, although 27γz forms typical round‐shaped PBs, whereas 16γz forms extensive thread‐like structures that irregularly enlarge the ER and remain in part soluble also in oxidizing conditions (Mainieri et al. 2018). Since disulphide bond‐mediated assembly into insoluble PBs is responsible for the retention of both 27γz and zeolin in the ER (Pompa and Vitale 2006; Mainieri et al. 2014), we verified whether the different traffic competences of zeolin and zeo2 can be related to differences in disulphide bond‐dependent polymerization (Figure 5, panels A‐C; panels D and E show quantification of the different assembly forms detected in panels A and B, respectively). Arabidopsis leaf extracts were prepared in the presence of non‐ionic detergent to solubilize the membranes of intracellular compartments, and subjected to velocity gradient sedimentation by ultracentrifugation. As previously found by expression in transgenic tobacco (Mainieri et al. 2004), in oxidizing conditions zeolin migrated with the pellet (P) at the bottom of the tube, indicating assembly into very large polymers with a molecular mass of at least several hundred thousand daltons (Figure 5C, fraction P), while the small amount of released PHSL (45 kD) migrated as correctly assembled trimers of around 150 kDa (Figure 5C, and see Mainieri et al. 2004). Intact unmodified zeo2 also sedimented in part with the pellet (Figure 5A, fraction P), but a relevant proportion of the polypeptides migrated in a region between the 66 and 160 kDa markers, with a peak around 160 kDa, suggesting assembly into trimers or tetramers (Figure 5A). The 20 kDa PHSL fragments co‐migrated roughly with the 240 kDa marker; this indicates possible hexamer formation upon fragmentation, a feature of 12S seed storage proteins: these are structurally closely related to 7S storage proteins, assemble into trimers in the ER and form hexamers after fragmentation into approximately two halves in storage vacuoles (Dickinson et al. 1989; Adachi et al. 2003). Under homogenation and ultracentrifugation in the presence of a reducing agent, zeolin PBs were disassembled into either monomers or dimers (Mainieri et al. 2004). The presence of reducing agents instead determined almost a complete recovery of zeo2, including its processed form, as trimers, while monomers were only faintly detected (Figure 5B, and compare quantifications in panels D and E). As expected, since PHSL does not contain cysteine residues, the reducing conditions did not affect the assembly of intact PHSL and PHSL fragments.

The band appearing around 30 kDa that was also detected in isopycnic gradients had a peculiar behaviour, being assembled into possible dimers (Figure 5A), which, however, highly polymerized or aggregated in reducing conditions (Figure 5B); this could suggest that this fragmentation product contains portions of both zein and PHSL, forming aggregates once one or more of its disulphide bonds are reduced. The exact nature of this polypeptide was not investigated further.

BiP was detected as a monomer in all samples, under both conditions (Figure 5A–C fractions 4–6), with a small proportion in the pellet fraction.

The results of assembly state analysis indicate that zeo2, unlike zeolin, does not undergo full, extensive polymerization. This may be the molecular feature of zeo2 allowing it increased intracellular traffic with respect to zeolin.

**FIGURE 6:**
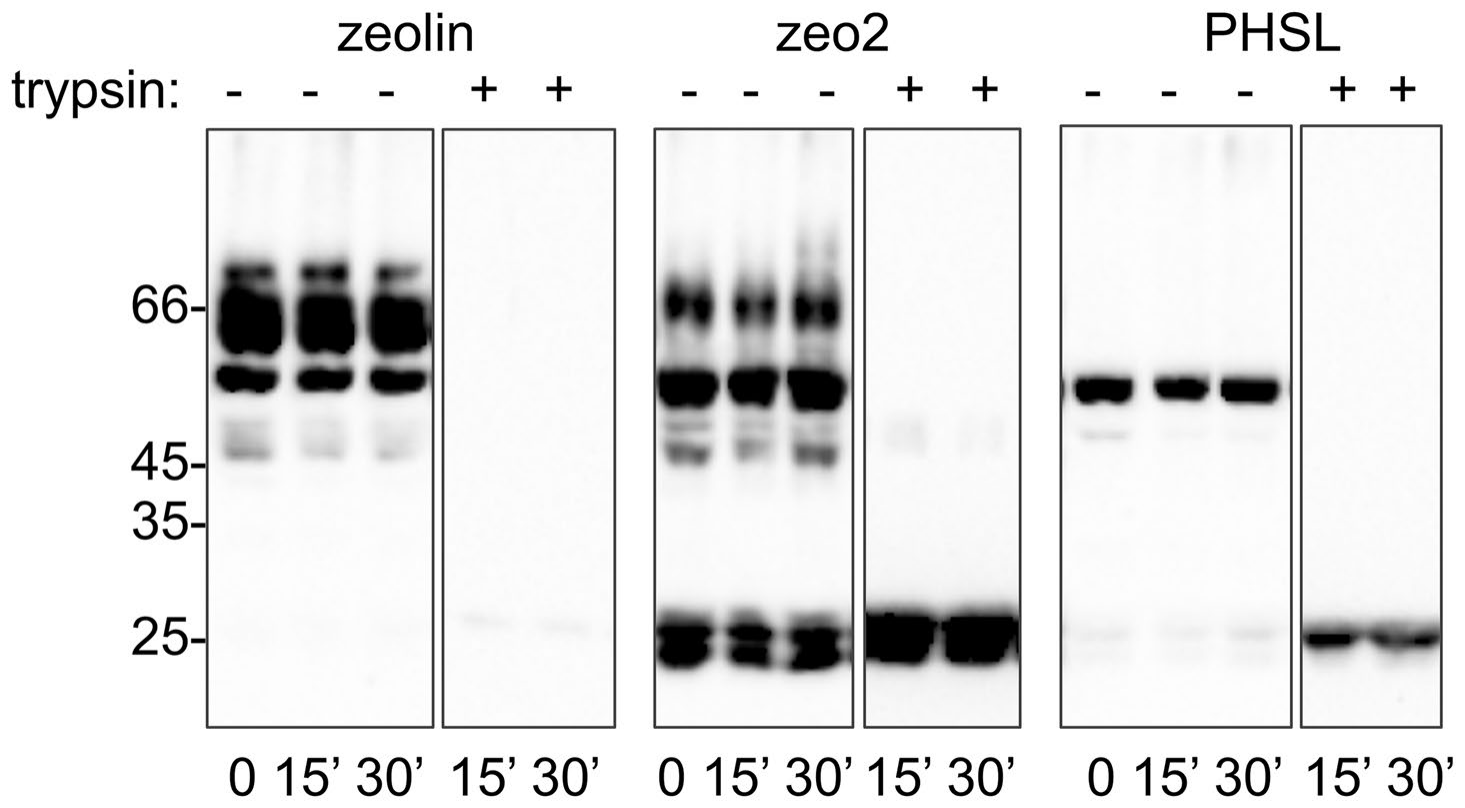
zeo2 trimers have features like those formed by natural PHSL. Total leaf homogenates from zeo2, zeolin or PHSL transgenic plants were supplemented with 10 μL of trypsin (+, from a 10 mg/mL stock solution prepared in 1 mM HCl) or with 10 μL of 1 mM HCl as a control (−) and subjected to digestion at 37°C for 0, 15, 30 min. Equal amounts of samples at different time points were analysed by SDS‐PAGE followed by western blot incubated with anti‐PHSL antibodies. Numbers on the left: SDS‐PAGE molecular mass markers (kDa).

Correctly assembled PHSL is cleaved in vitro by trypsin into stable fragments similar in size to those produced upon its delivery to vegetative vacuole, whereas unassembled PHSL monomers are fully degraded by the enzyme (Ceriotti et al. 1991). In vitro trypsin digestion of extracts from the arabidopsis plants producing zeolin, zeo2 or PHSL was therefore performed to have more information on the conformation of zeo2 (Figure 6). PHSL expressed in arabidopsis produced the expected fragments. Intact zeolin and zeo2 were not detectable already after 15 min trypsin digestion. Almost no PHSL fragment was detectable by the anti‐PHSL antiserum upon zeolin digestion, indicating full digestion of its PHSL portion, whereas treatment of zeo2 markedly increased the amount of PHSL fragments. These results confirmed that the PHSL sequence of zeo2 efficiently drives the formation of trimers with characteristics similar to those of natural PHSL, while in zeolin the dominant, fast polymerization driven by the cysteine residues of the 27γz N‐terminal sequence largely inhibits trimerization.

**FIGURE 7:**
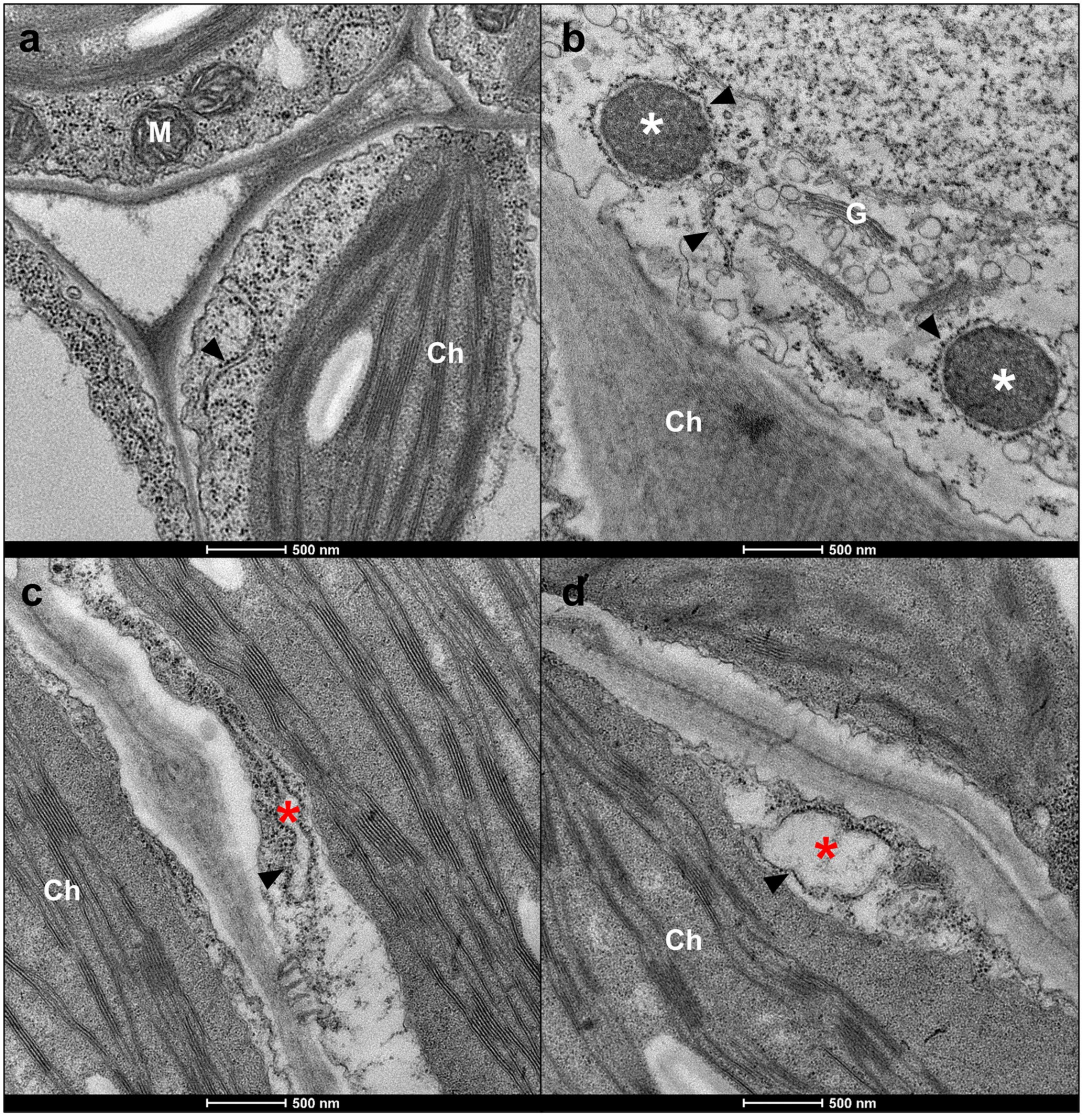
The presence of zeo2 alters the ER morphology. Ultrathin sections were prepared from leaves of 6‐week‐old arabidopsis plants Col‐0 (a) or expressing zeolin (b) or zeo2 (c, d) were analysed. After post‐fixing with osmium, the sections were observed at the transmission electron microscope. Black arrows in a, b, c, d: Ribosomes attached on the cytosolic side of the ER membrane. White asterisks (b): Zeolin protein bodies. Red asterisks (c, d): Zeo2 enlarged ER lumen. Ch, chloroplasts; M, mitochondria; G, Golgi apparatus. Scale bars 500 nm.

**FIGURE 8:**
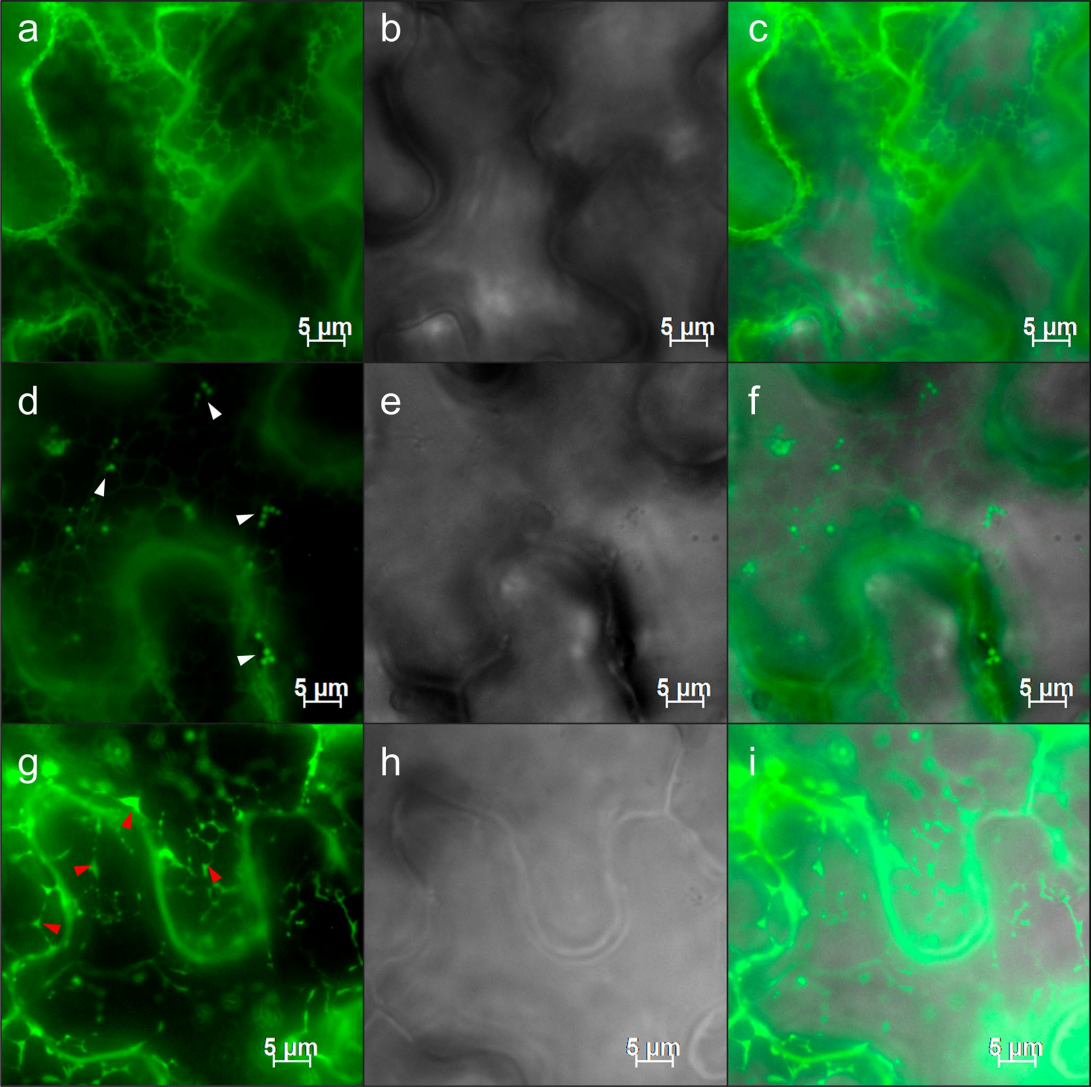
Zeo2 remodels the endoplasmic reticulum but does not form canonical protein bodies. *N. tabacum* leaves stably expressing the ER luminal marker GFP‐HDEL were transiently transfected with agrobacteria carrying plasmids encoding zeolin (d, e, f) or zeo2 (g, h, i), or mock transfected (a, b, c). Two days after infiltration, leaf fragments were analysed by fluorescence microscopy. GFP‐HDEL fluorescence was detected with BP 470/20‐BP 505–530 ex‐em filter set (a, d, g). Brightfield (b, e, h) and merge of fluorescence and brightfield (c, f, i). White arrowheads indicate protein bodies (PBs) formed by zeolin. Red arrowheads indicate brighter ER structures formed by zeo2. Both PBs and brighter ER structures contain GFP‐HDEL. Bar: 5 μm.

Subcellular fractionation indicated that, despite its increased traffic competence, a relevant amount of zeo2 can be detected in ER‐derived microsomes (Figure 3). We thus analysed whether this has an effect on ER morphology. Electron microscopy analysis showed that in leaf cells of transgenic arabidopsis expressing zeo2 the ER lumen often had enlarged regions (Figure 7, red asterisks in panels c and d, and compare with plants expressing the empty plasmid in panel a). These enlargements contained very little electron dense material and were clearly different from the round‐shaped, highly electron dense PBs formed in plants expressing zeolin (Figure 7, white asterisks in panel b). We also transiently expressed by agroinfiltration zeolin or zeo2 in leaves of transgenic tobacco plants constitutively expressing the ER marker GFP‐HDEL. Fluorescence microscopy confirmed the presence of enlarged ER areas highlighted by GFP‐HDEL and a remodelling of the ER morphology when zeo2 was expressed (Figure 8, panels g‐i, red arrowheads, and compare with the canonical ER structure in mock transfected leaves in panels a‐c). As expected by the results of electron microscopy, numerous round‐shaped fluorescent PBs were instead detected upon transient expression of zeolin (Figure 8, panels d‐f, white arrowheads).

Analysis of ER morphology thus confirmed that zeo2 is unable to form PBs and indicated it causes ER enlargements; however, these effects were less drastic than those of 16γz (Mainieri et al. 2018) since the enlargements were much less pronounced and almost no electron‐dense material was visible.

### 3.4 The Destiny of zeo2 Does Not Depend on Disulphide Bond Formation

**FIGURE 9:**
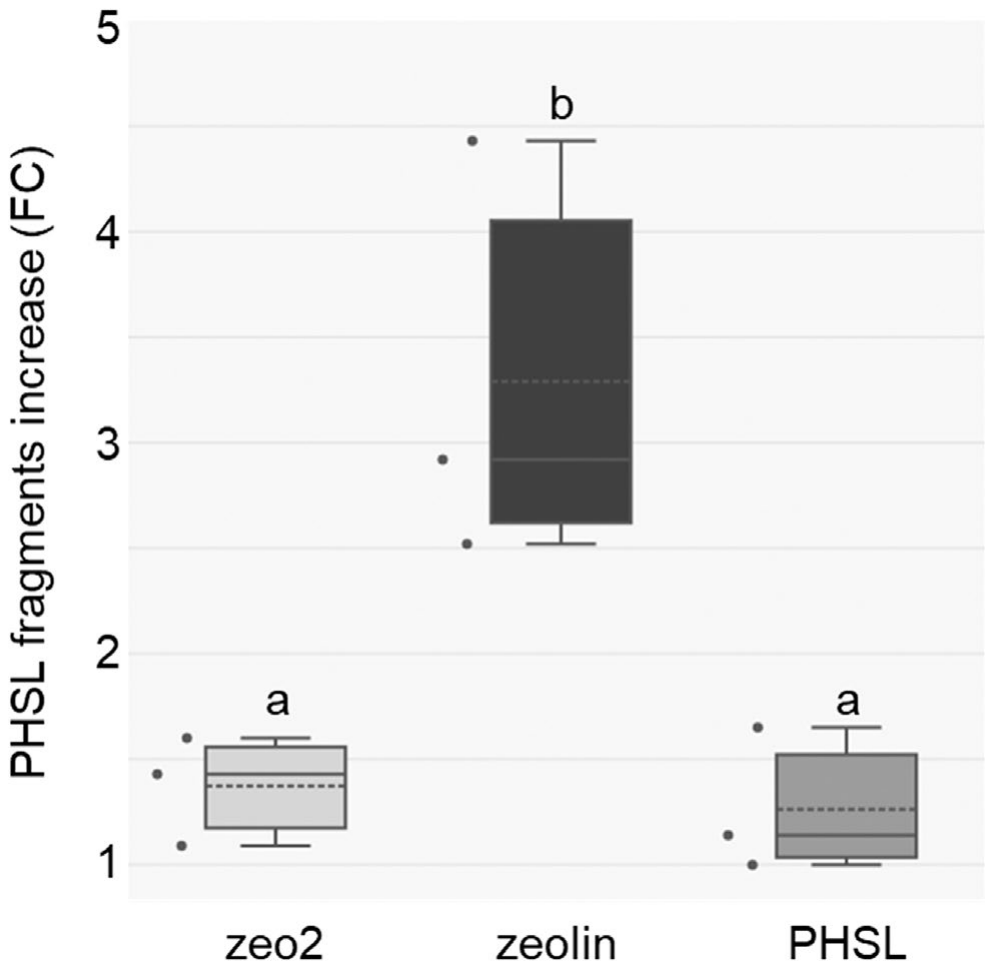
In vivo treatment with reducing agent promotes traffic to the vacuole of zeolin, but not those of zeo2 and PHSL. Seedlings from zeo2, zeolin or PHSL transgenic plants were incubated for 24 h in the presence (+DTT) or absence (co) of 2 mM DTT in water. Proteins were extracted and equal amounts were analysed by SDS‐PAGE and protein blot using anti‐PHSL antiserum. The PHSL fragments in zeolin, zeo2 or PHSL samples were quantified by densitometry of their specific protein blot bands, relative to the filter background, and expressed as percentage of the total amount of the detected polypeptides in the lane. The fold change in the percentages of PHSL fragments in DTT‐treated on the untreated samples (% + DTT/% −DTT) was calculated. Data are from three fully independent biological replicates. Statistical significance was determined by one‐way ANOVA followed by post hoc Tukey HSD test. Values with different letters are significantly different (*p* < 0.05). Dots: Individual data points. Dashed and solid line in the box: Position of mean and median, respectively.

Reducing agents are inhibitors of protein synthesis, disulphide bond formation and traffic, as well as UPR‐mediated inducers of ER folding factors. Despite these effects, in vivo treatment with reducing agents increased the solubility of newly synthesized zeolin and its intracellular traffic, resulting in both secretion and increased vacuolar delivery, indicating that disulphide‐dependent polymerization, not quality control interactions, is a major determinat for zeolin ER retention (Pompa and Vitale 2006). Zeo2 assembly and traffic instead suggest that disulphide bonds play a minor role in its destiny. We therefore incubated seedlings expressing zeo2, zeolin or PHSL for 24 h in the presence or absence of low concentration of the reducing agent dithiothreitol and analysed the electrophoretic banding pattern of the three recombinant proteins (Figure 9). Quantification of protein blot bands indicated that the reducing agent markedly increased the relative amount of PHSL fragments originating from zeolin, confirming its increased traffic, but had no significant effect on fragmentation of zeo2 and PHSL (it should be remembered that there are no Cys residues in PHSL).

The failure of the reducing agent to stimulate zeo2 fragmentation further supports the indication that the disulphide‐dependent formation of traffic‐incompetent polymers, which is a major determinant for zeolin destiny, does not occur to a significant extent in zeo2.

### 3.5 Zeo2 Is More Rapidly Digested Than Zeolin During Germination

**FIGURE 10:**
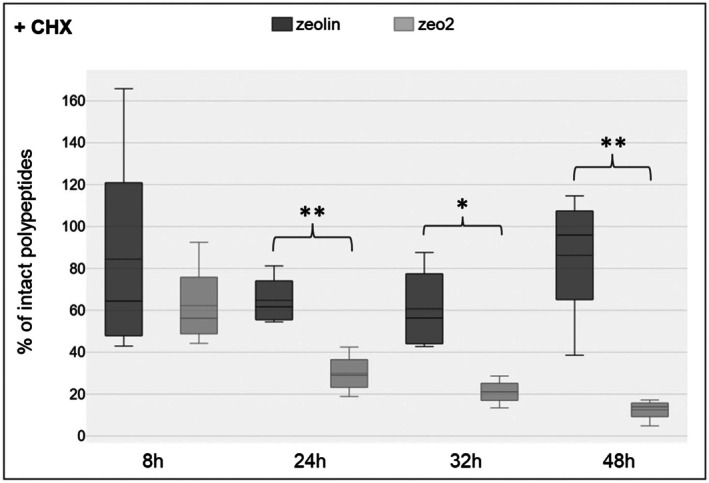
Zeo2 is more subjected to proteolysis than zeolin during seed germination. Twenty zeolin or zeo2 dry seeds were time‐course germinated in water in the presence of 100 μM cycloheximide. At each time‐point, germinating seeds were homogenized, and equal amounts of each homogenate were analysed by SDS–PAGE, followed by protein blot with anti‐PHSL antibodies. Total proteins were stained with Ponceau S to visualize RbcL. The total amounts of intact zeolin and zeo2 polypeptides were quantified by densitometry of their specific protein blot bands (Figure S5), normalized to the amounts of RbcL, and expressed as percentage (%) relative to the amounts at 0 h = 100%. Data are from four independent experiments. Statistical significance was determined by pairwise *T*‐test with post hoc Tukey HSD test at each time point. *p < 0.05; **p < 0.01. Dashed and solid line in the box: Position of mean and median, respectively.

Since seed storage proteins function as a source of amino acids for the new growing plant, they have evolved to accumulate during seed development but at the same time to be efficiently broken down upon germination (Tan‐Wilson and Wilson 2012). However, the individual rates of mobilization vary, possibly being influenced by the protein solubility and subcellular localization, characteristics of the different proteases involved as well as the presence of companion storage proteins (Torrent et al. 1989; De Barros and Larkins 1990). Given the different solubility and traffic competences of zeo2 and zeolin, we have thus compared the rate of their mobilization during germination. As the two recombinant DNA coding sequences were under the control of a constitutive promoter, germination was performed in the presence of the protein synthesis inhibitor cycloheximide to inhibit zeo2 and zeolin neosynthesis. At each germination time‐point, germinating seeds were homogenized and equal amounts of each homogenate were analysed by SDS–PAGE, followed by protein blot with anti‐PHSL antibodies. The total amounts of intact zeolin and zeo2 polypeptides were quantified by densitometry of their specific protein blot bands, normalized to the amounts of RbcL, and expressed as percentage (%) relative to the intact protein amounts at 0 h = 100%. The percentage of intact zeolin did not change significantly during the first 48 h of germination, while that of zeo2 rapidly decreased (Figure 10, and see Figure S5 for protein blot images), indicating that zeo2 is more susceptible than zeolin to the hydrolases that degrade storage proteins during arabidopsis germination.

## 4 Discussion

A natural zein PB is a very large heteropolymer in which the different zein classes (α, β, γ, δ) are orderly distributed (Holding 2014; Pedrazzini et al. 2016). When mutations lead to strong reduction or absence of one class, the PB size or shape is altered to various extents, often compromising the whole grain texture (Holding 2014). Not all zein polypeptides however have the same importance in determining formation of a PB. Thus, 27γz has a major role in initiating natural PB formation (Guo et al. 2013), wheras the much less abundant 16γz seems to function at the contacts between 27γz and the inner PB core mainly constituted by α‐zeins (Kim et al. 2002; Mainieri et al. 2018).

The features of 27γz have first led researchers to identify its N‐terminal domain as the determinant for its retention into the ER (Geli et al. 1994) and then to test whether the identified domain can be used in biotechnology for high protein accumulation. The first chimeric construct made was zeolin (Mainieri et al. 2004), because of both the high importance of phaseolin as a legume storage protein and of its structural characteristics: the single N‐linked glycan can be used as a marker for traffic through the Golgi complex, and fragmentation in vegetative vacuoles as a marker for intracellular traffic.

We have now shown here that zeo2, a chimeric construct where the 27γz domain of zeolin was substituted by the 16γz paralog domain, has a markedly different behaviour with respect to zeolin. Zeo2 does not form PB and in relevant part traffics to the vacuole via the Golgi complex. The destiny of zeo2 is also distinct from that of ectopically expressed 16γz, which assembles into large thread‐like structures that remain in the ER (Mainieri et al. 2018). The destiny of zeo2 seems therefore mainly determined by its phaseolin portion, whereas the natural albumin‐like 16γz C‐terminal domain has no dominant effect: 2S albumins are monomeric, soluble vacuolar proteins but 16γz is an ER resident polymeric protein also when expressed alone. The observation that zeo2 has less extensive interactions with BiP and stimulates synthesis of this major ER chaperone to lesser extents compared to zeolin, whereas 16γz is instead a stronger BiP ligand and inducer than 27γz (Brocca et al. 2021), also indicates that phaseolin, not the 16γz domain, mainly determines folding and assembly of zeo2. Since phaseolin monomers also interact with BiP, but the rapid trimerization masks their BiP binding sites (Vitale et al. 1995), it can be hypothesized that rapid zeo2 trimerization also abolishes possible interactions between BiP and the 16γz domain and reduces, but not fully abolishes, the probability that homotypic interactions driven by this domain lead to the formation of insoluble polymers.

Absence of extensive, prolonged interactions with BiP, the presence of the PHSL Asn‐linked oligosaccharide chain and the scarce ability to form insoluble PBs must facilitate traffic of a large proportion of zeo2 molecules from the ER along the secretory pathway. Fragmentation of PHSL into two approximate halves is an indicator of its sorting to vegetative vacuoles, as mutated PHSL lacking its C‐terminal propetide AFVY is secreted and does not undergo extracellular fragmentation (Frigerio et al. 1998). Vacuolar sorting of zeo2 that traffics from the ER seems therefore rather efficient. It seems unlikely that this reflects misfolding and delivery to the vacuole, which could be an alternative quality control route to dislocation from the ER to the cytosol (Foresti et al. 2003; Pimpl et al. 2006), since at least the phaseolin portion of these zeo2 molecules correctly forms trimers. This is also supported by in vitro trypsin digestion: zeo2 and PHSL are similarly cleaved, giving rise to stable phaseolin fragments, whereas the phaseolin domain of zeolin is fully degraded. Therefore, phaseolin trimerization is inhibited by the 27γz domain, which has dominant PB‐forming activity dependent on inter‐chain disulphide bonds, but not by the 16γz domain, which has a lower number of Cys residues and amphipathic repeats. The less important role of disulphide bonds in determining the destiny of zeo2 compared to zeolin is also supported by the results of in vivo treatment with a reducing agent, which stimulates traffic to the vacuole of zeolin but has no marked effect on both zeo2 and PHSL. Vacuolar delivery of zeo2 could be due to the PHSL C‐terminal sorting signal, which is however internal in the zeo2 primary sequence, or some other structural feature of zeo2. Transient polymerization driven by the 16γz domain could also play a role in zeo2 vacuolar sorting, as suggested by a number of other observations: (i) PHSL, but not its secreted mutated form, undergoes transient polymerization during its traffic to the vacuole (Castelli and Vitale 2005), (ii) addition of a C‐terminal Cys residue to the secreted form results in the formation of an inter‐chain disulphide bond and partial vacuolar delivery (Pompa et al. 2010), (iii) progressive substitution of cysteine residues of the N‐terminal domain of 27γz with serine residues leads to partial vacuolar delivery, only full substitution leading to very efficient secretion (Mainieri et al. 2014). Zeo2 can therefore be used to have more insights on the not yet clearly known interactions that lead to efficient storage protein sorting to vacuoles (Di Sansebastiano et al. 2018) and the evolutionary relationships between storage protein sorting to vacuoles and PB formation (Pedrazzini et al. 2016).

### 4.1 Implications for the Nutritional Improvement of Seed Storage Proteins

Improvement of the essential amino acid content of seed storage proteins through protein engineering has been an aim since the dawn of transgenic plant technology (Hoffman et al. 1988) but, after decades, the results are still not satisfactory (Galili and Amir 2013; Amir et al. 2019). A major problem is that designing storage protein mutations or neoproteins with planned optimal amino acid content has often resulted in protein instability, probably due to misfolding and therefore degradation by the ER quality control system (Ohtani et al. 1991, Nuttall et al. 2003), missorting (Alvarez et al. 1998) or scarce resistance in storage vacuoles (Hoffman et al. 1988; Zhang et al. 2014).

Unlike the products of other attempts to improve the amino acid content of phaseolin (Hoffman et al. 1988, Nuttall et al. 2003), zeolin is a rather stable protein (Mainieri et al. 2004). Zeo2 accumulates to lower amounts compared to zeolin, but the difference does not seem to be very marked. Phaseolin has three Met and no Cys residues. The 16γz N‐terminal domain used to produce zeo2 adds one Met and three Cys residues, whereas the 27γz domain of zeolin adds six Cys and no Met residues. Remarkably, zeo2 accumulates to clearly higher amounts than phaseolin in transgenic Arabidopsis, and the difference seems to be mainly due to the fraction of zeo2 that remains intact in the ER lumen (see Figure 2). Zeo2 accumulated in seeds has also higher susceptibility than zeolin to degradation during Arabidopsis seed germination, which is a desired feature of seed storage proteins and may be due to the proportion of zeo2 that reaches storage vacuoles. Thus, the double destiny of zeo2, which in part remains in the ER and in part is sorted to storage vacuoles, could constitute a positive feature for its exploitation as a nutritionally improved protein. It should however be noticed that this is valid for the many plants that accumulate seed storage proteins in vacuoles. Cereal prolamins are instead degraded by proteases secreted by the aleurone layer, since the endosperm is a dead tissue at seed germination. We therefore do not know whether the difference between zeolin and zeo2 mobilization would be observed also in germinating cereal seeds.

## Author Contributions

E.P. and A.V. conceived the study and wrote the manuscript; E.P. designed and performed the experiments and analysed all the data; E.P, E.C., L.L., M.T. conducted the experimental work.

## Funding

This work was funded by the Next Generation EU, Piano Nazionale di Ripresa e Resilienza‐PNRR Mission 4 Component 2 Investment 3.1 ITINERIS IR0000032, CUP B53C22002150006 and Next Generation EU, Piano di Ripresa e Resilienza‐PNRR Mission 4 Component 2 Investment 1.4 AGRITECH CN00000022, CUP B83C22002840001 (to E.P.).

## Conflicts of Interest

The authors declare no conflicts of interest.

## Supporting information


**Figure S1:** Alignment of zeolin and zeo2 protein sequences. Zeolin: upper sequence (see Mainieri et al. 2004). Zeolin 2 (lower sequence). Grey: mature PHSL sequence (AA 25–421, Pedrazzini et al. 1997) and synthetic linker (AA 422–436, Mainieri et al. 2004). Green: cotranslationally removed PHSL N‐terminal signal peptide (AA 1–25). Yellow: N‐terminal domains of 27γz (AA 437–525, Mainieri et al. 2004) and 16γz (AA 437–482, Mainieri et al. 2018), starting from the fifth amino acid after their respective signal peptide cleavage sites. Cys and Lys residues are in red and blue, respectively. Cys residues conserved between zeolin and zeolin 2 are boxed.

**Figure S2:** Zeo2 does not stress the ER folding machinery. 20‐days old seedlings of zeo2, zeolin, PHSL transgenic lines and Col‐0 were homogenized with five volumes of homogenization buffer and equal amounts of total proteins were analysed by SDS–PAGE, followed by protein blot with anti‐BiP antibodies. BIP amounts were quantified by densitometry of the specific protein blot band in different samples and expressed as protein fold change relative to the Col‐0 plant (Col‐0 = 1). Data are from two fully independent biological replicates (biol rep 1 and biol rep 2).

**Figure S3:** Zeo2 does not form high‐dense PBs, remaining available for intracellular traffic. Distribution of zeolin (A, B), zeo2 (C, D) or BiP (A–F) polypeptides in the isopycnic gradient fractions shown in Figure 3. Polypeptides were analysed by densitometry of their specific protein blot bands and expressed as percentage (%) on total protein in the blot.

**Figure S4:** Zeo2 traffic to the vacuole passing through the Golgi complex. Longer exposure of protein blot in Figure 4.

**Figure S5:** Zeo2 is more digestible than zeolin during germination. Blot images of one representative replicate used in the quantifications shown in Figure 8. The positions of zeolin (A), zeo2 (B), PHSL and of the PHSL fragments (A, B) are indicated. Numbers on the left: molecular mass markers in kDa.

## Data Availability

The data that supports the findings of this study are available in the main text and in the Supporting Information of this article.
