# Supplementary figures and images for: A Fusion Between Common Bean Phaseolin and the N‐Terminal Domain of Maize 16 kDa Gamma Zein: Clues for Designing Nutritionally Improved Seed Storage Proteins
Source: Plant Biotechnol J. 2026 Jul 5:10.1111/pbi.70719. Online ahead of print. doi: 10.1111/pbi.70719 (PMC13399306; doi:10.1111/pbi.70719)

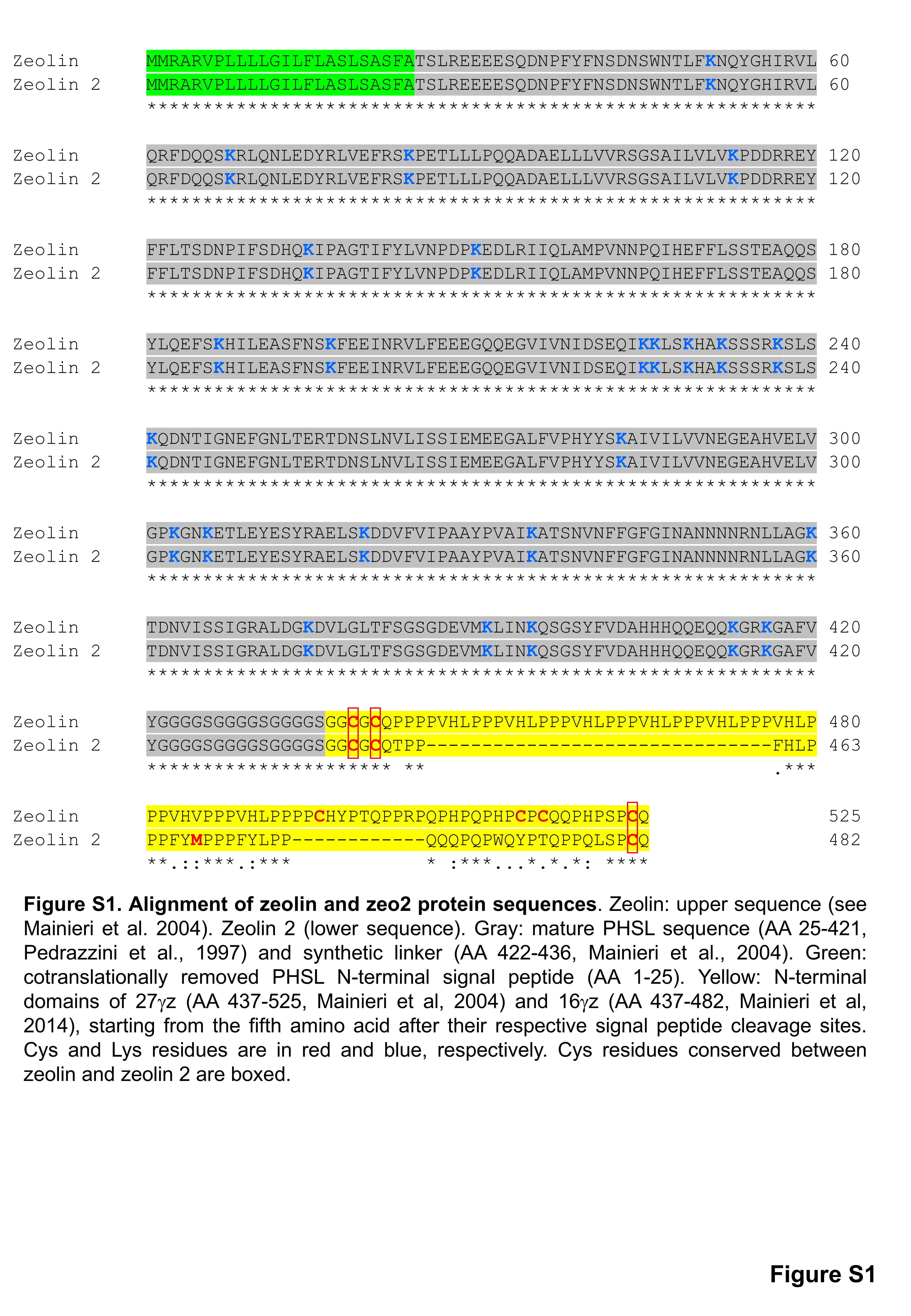

Supplement: Supplementary file 1 — Figure S1: Alignment of zeolin and zeo2 protein sequences. Zeolin: upper sequence (see Mainieri et al. 2004). Zeolin 2 (lower sequence). Grey: mature PHSL sequence (AA 25–421, Pedrazzini et al. 1997) and synthetic linker (AA 422–436, Mainieri et al. 2004). Green: cotranslationally removed PHSL N‐terminal signal peptide (AA 1–25). Yellow: N‐terminal domains of 27γz (AA 437–525, Mainieri et al. 2004) and 16γz (AA 437–482, Mainieri et al. 2018), starting from the fifth amino acid after their respective signal peptide cleavage sites. Cys and Lys residues are in red and blue, respectively. Cys residues conserved between zeolin and zeolin 2 are boxed. [file PBI-9999-0-s003.tif]

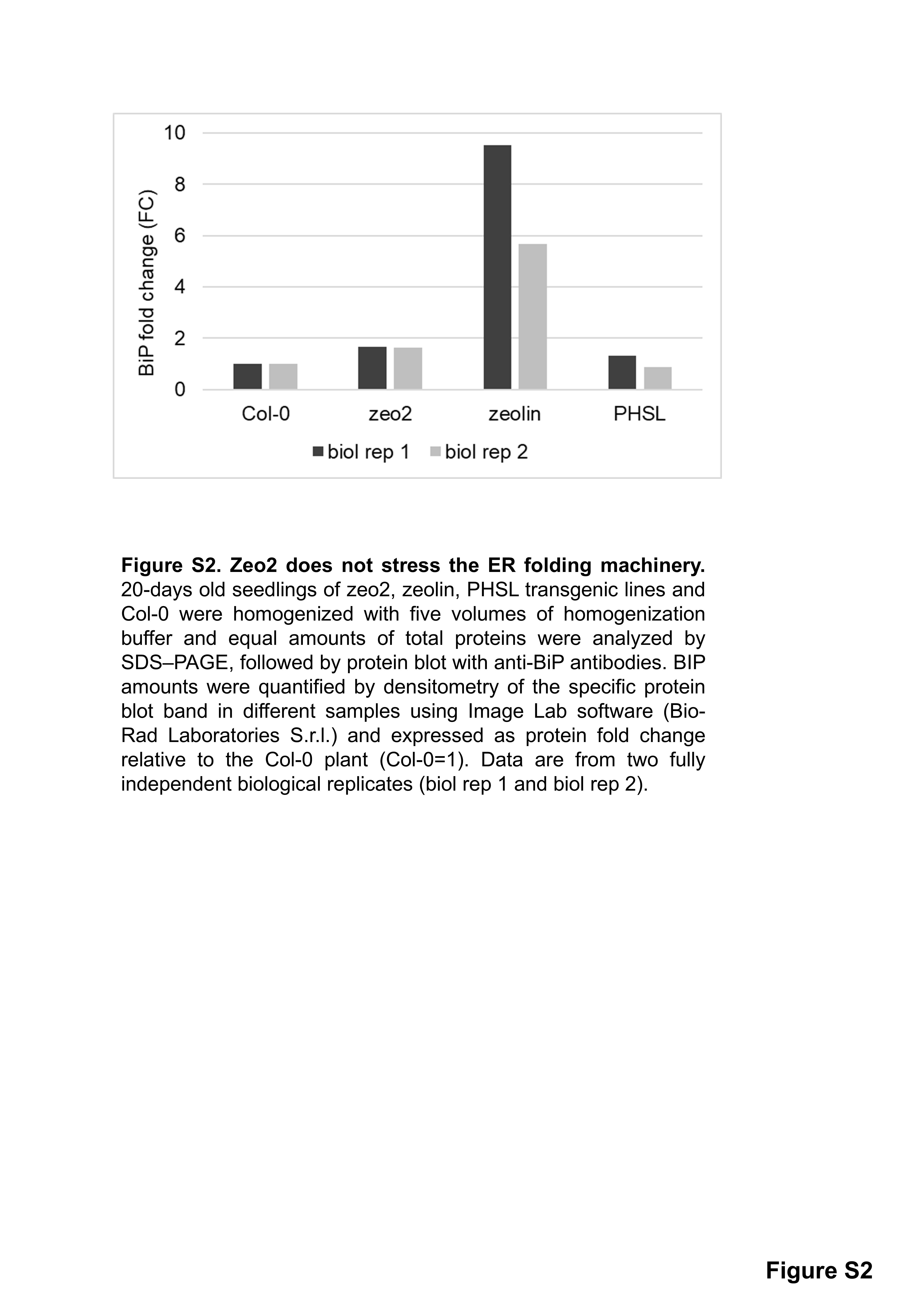

Supplement: Supplementary file 2 — Figure S2: Zeo2 does not stress the ER folding machinery. 20‐days old seedlings of zeo2, zeolin, PHSL transgenic lines and Col‐0 were homogenized with five volumes of homogenization buffer and equal amounts of total proteins were analysed by SDS–PAGE, followed by protein blot with anti‐BiP antibodies. BIP amounts were quantified by densitometry of the specific protein blot band in different samples and expressed as protein fold change relative to the Col‐0 plant (Col‐0 = 1). Data are from two fully independent biological replicates (biol rep 1 and biol rep 2). [file PBI-9999-0-s004.tif]

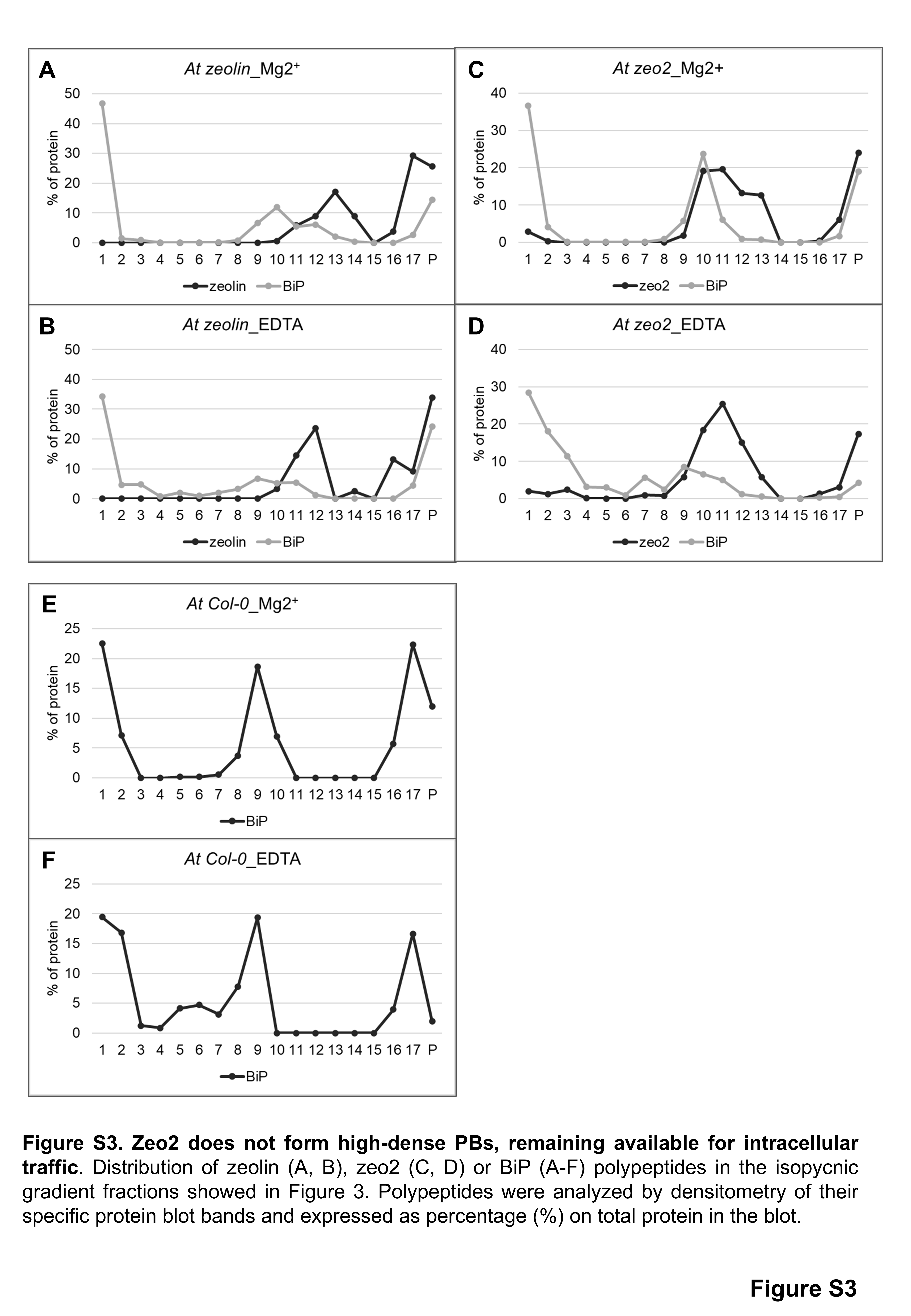

Supplement: Supplementary file 3 — Figure S3: Zeo2 does not form high‐dense PBs, remaining available for intracellular traffic. Distribution of zeolin (A, B), zeo2 (C, D) or BiP (A–F) polypeptides in the isopycnic gradient fractions shown in Figure 3. Polypeptides were analysed by densitometry of their specific protein blot bands and expressed as percentage (%) on total protein in the blot. [file PBI-9999-0-s001.tif]

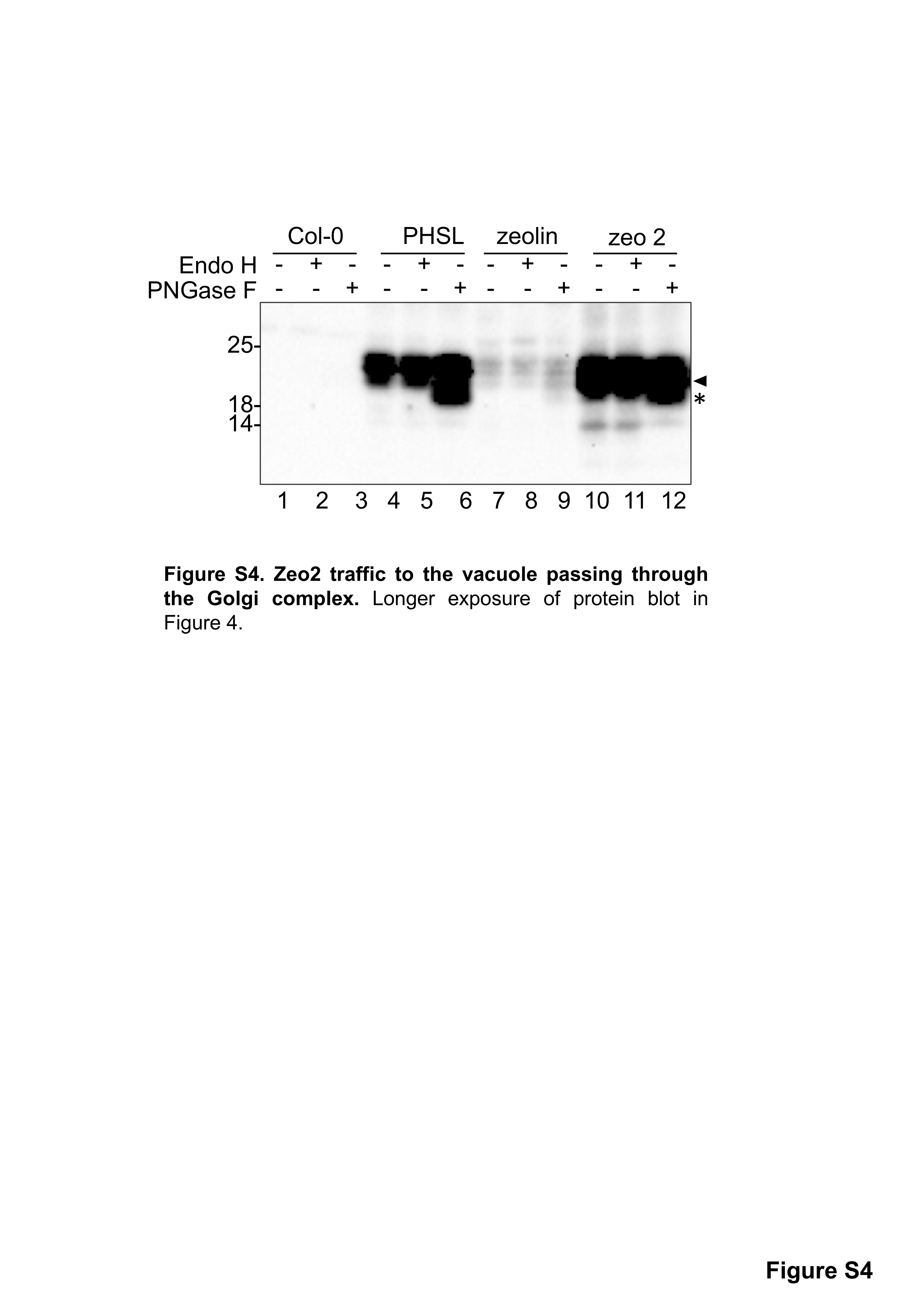

Supplement: Supplementary file 4 — Figure S4: Zeo2 traffic to the vacuole passing through the Golgi complex. Longer exposure of protein blot in Figure 4. [file PBI-9999-0-s005.tif]

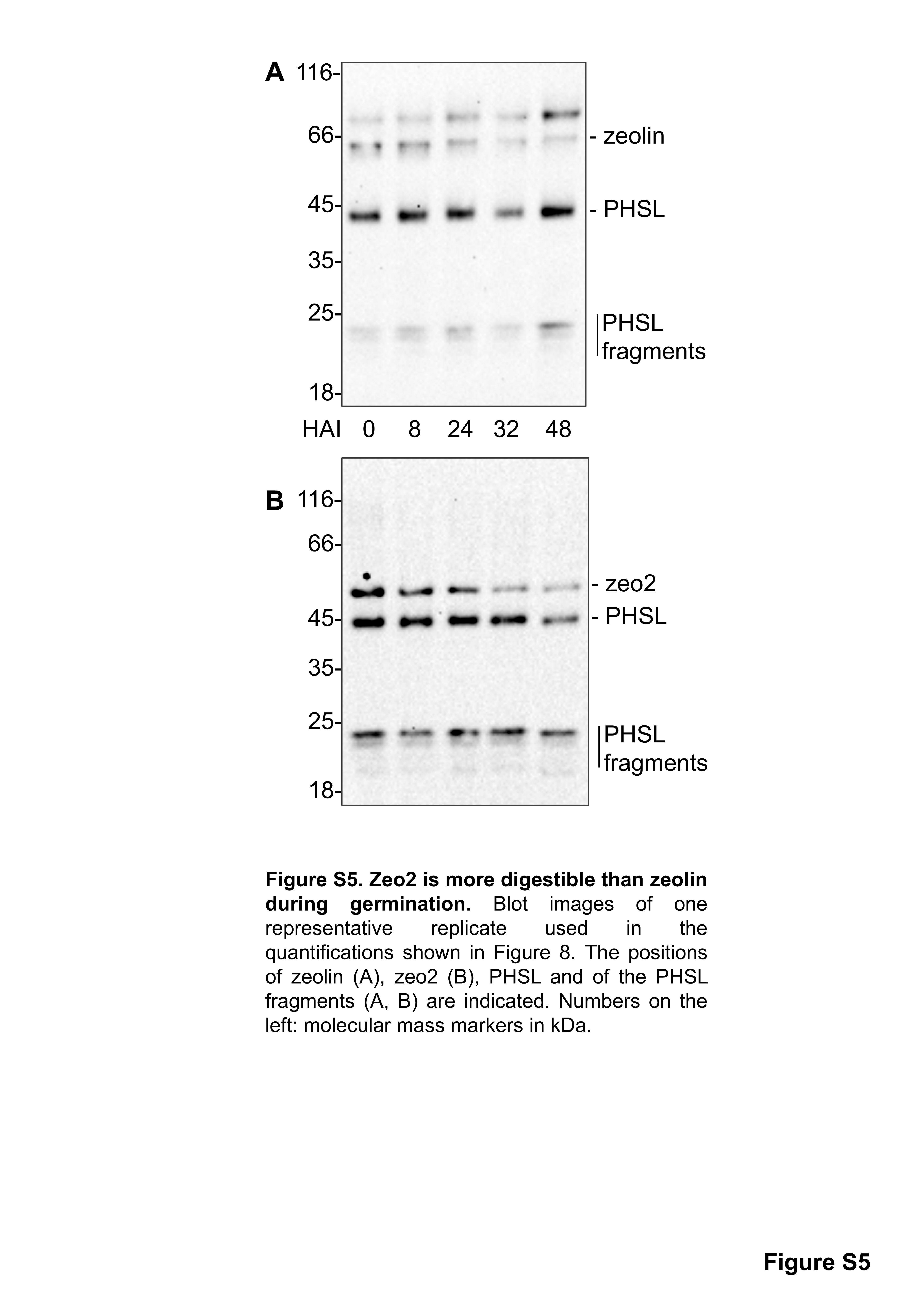

Supplement: Supplementary file 5 — Figure S5: Zeo2 is more digestible than zeolin during germination. Blot images of one representative replicate used in the quantifications shown in Figure 8. The positions of zeolin (A), zeo2 (B), PHSL and of the PHSL fragments (A, B) are indicated. Numbers on the left: molecular mass markers in kDa. [file PBI-9999-0-s002.tif]
